# An Extract of Olive Mill Wastewater Downregulates Growth, Adhesion and Invasion Pathways in Lung Cancer Cells: Involvement of CXCR4

**DOI:** 10.3390/nu12040903

**Published:** 2020-03-26

**Authors:** Matteo Gallazzi, Marco Festa, Paola Corradino, Clementina Sansone, Adriana Albini, Douglas M. Noonan

**Affiliations:** 1 Department of Biotechnology and Life Sciences, University of Insubria, 21100 Varese, Italy; m.gallazzi7@studenti.uninsubria.it (M.G.); douglas.noonan@uninsubria.it (D.M.N.); 2 Laboratory of Vascular Biology and Angiogenesis, Science and Technology Pole (PST), IRCCS MultiMedica, 20138 Milan, Italy; marco.mg.festa@gmail.com (M.F.); paola_corradino@yahoo.it (P.C.); 3 Marine Biotechnology Department, Stazione Zoologica Anton Dohrn, 80121 Naples, Italy; clementina.sansone@szn.it; 4 School of Medicine and Surgery, University of Milano-Bicocca, 20900 Monza, Italy

**Keywords:** Lung cancer, inflammation, angiogenesis, polyphenols, hydroxytyrosol, migration, chemoprevention, nutraceutical, STAT3, chemokines, CXCL12, olive oil

## Abstract

Several diet-derived compounds have been reported to exert antioxidant, anti-proliferative and anti-angiogenic effects in numerous cancers and could be beneficial in cancer prevention. Olive oil production involves the generation of an aqueous phase defined as olive mill wastewater (OMWW), a polluting effluent rich in soluble polyphenols. Here, we assessed the cancer preventive properties exerted by a purified extract of OMWW (A009) for its activity on lung cancer cell lines. Hydroxytyrosol, the most abundant polyphenol present in our A009 extracts, was used as reference molecule in the assays performed. Extracts from OMWW from two different olive oil cultivars were used. We found that the A009 extracts limit lung cancer cell proliferation in a dose and time dependent manner. These effects were associated with the induction of apoptosis. A009 extracts were effective in inhibiting adhesion capabilities on a fibronectin layer accompanied with a reduction in their ability to generate invasive sprouts in a Matrigel layer. The production of chemokine CXCL12 and CXCR4 receptor were reduced by treatment with the extracts. Also, A009 interfered with the production of proangiogenic and pro-inflammatory VEGF, CXCL8, and CCL2 (as detected by FACS analysis) in the lung cell lines. A009 extracts were able to decrease STAT3 phosphorylation in lung cancer cells. Our results show that A009 extracts reduced activities related to tumor cell behavior in lung cancer cell lines, suggesting that they could have a potential cancer preventive role.

## 1. Introduction

Lung cancer is by far the leading cause of cancer death worldwide [1], the predominant form is non-small cell lung cancer (NSCLC), comprising 75–80% of cases [2], which is divided into two main subtypes, adenocarcinomas (40%) and squamous cell carcinomas (15–25%). Patients diagnosed at an early stage (stage I and II NSCLC) have a better prognosis (60–80% survival at five years), while representing only 20–25% of all NSCLC diagnosed.

After the success in melanoma and hematological malignancy, immunotherapy has now emerged as a promising approach also in lung cancers. However, as for chemo, radio, and targeted therapy, there are some limitations in success due to the induction of resistance and/or the manifestation of side effects. In this complex scenario, prevention still remains the most promising approach on all cancer types, including lung.

Abstention from smoke and tobacco use, reduction of exposure to pollutants, a correct dietary regimen and avoidance of a sedentary lifestyle, are all part of a correct preventive setting. The Mediterranean diet has been demonstrated to positively impact on human health and the beneficial effects of a Mediterranean diet regimen, together with physical activity, are largely recognized as protective, ranging from cardiovascular and neurological disorders to metabolic syndrome and cancers. All these disorders share chronic inflammation and oxidative damage as relevant features.

Olive oil represents a basic component of the Mediterranean diet [3,4,5] and several data from both observational and clinical studies showed that the consumption of olive oil is associated with reduced risk for chronic-degenerative disease. More than 30 different phenolic compounds have been determined in extra virgin olive oil (EVOO), making this condiment an extraordinary dietary source of anti-oxidant agents [6].

Hydroxytyrosol (HT) represents the most abundant polyphenol present in olive oil and its beneficial properties, including anti-oxidant [7], anti-apoptotic [8], anti-tumor [3,9,10,11,12], and anti-inflammatory activities [18], have been largely demonstrated in several in vitro and in vivo studies. Olive oil production generates large volumes of waste material, whose management strongly impacts on production costs and represents a relevant source of environmental pollutants [13,14]. A proprietary method has been used to recover part of the wastewater through filtration and purification for further use. The chemical characterization of these OMWW extracts revealed that they are largely enriched in soluble polyphenols [17]. Recovering polyphenols from waste material is an approach that we have now established, and the ultra-purified extracts from OMWWs that we termed A009 [10,15,16] have been tested for effects on cancer cells. Here, we explored the potential chemoprevention associated activities of these polyphenol-rich extracts in in vitro models of lung cancer and explored their molecular mechanisms.

## 2. Materials and Methods 

### 2.1. Reagents and Chemicals

**Figure 1:**
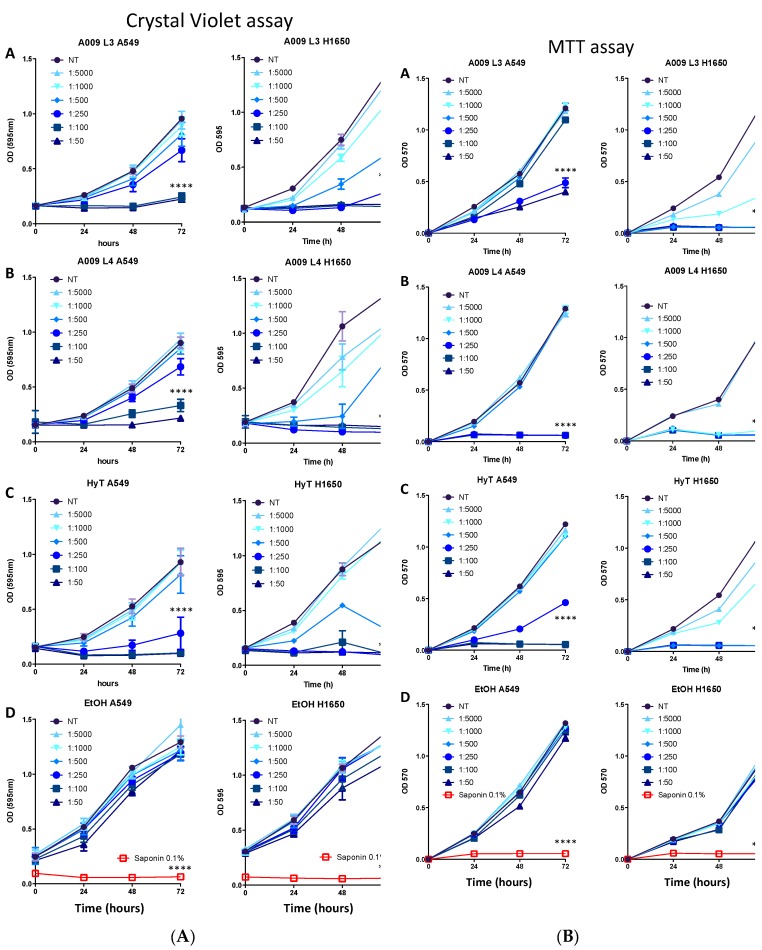
(**A**) Effect of the A009 extracts on lung cancer cell proliferation in vitro. Lung carcinoma cell lines A549 (A-D) and H1650 (A-D) were treated for 24-48-72 hours with 2 different batches of OMWW A009 extracts (L4 and L3), at dilution range 1:5000 to 1:50, or with hydroxytytosol (HyT) at the same concentration present in the dilutions of A009. The effect on cell proliferation was assessed using the Crystal Violet colorimetric assay. At each time point the absorbance value at 595 nm was detected. The data are shown as a percentage of proliferating cells, per experimental condition. The 0.1% saponin detergent was used as a positive control, ethanol was used as a negative control for HyT. Results are shown as mean ± SEM, two-way ANOVA, *** *p* < 0.001, **** *p* <  0.0001. (**B**) Effect of the A009 extracts on lung cancer cell proliferation in vitro. Lung carcinoma cell lines A549 (A–D) and H1650 (A–D) were treated for 24-48-72 hours with 2 different batches of A009 extracts (L4 and L3), at dilution range 1:5000 to 1:50, or with hydroxytytosol (HyT) at the same concentration present in the dilutions of A009. The effect on cell proliferation was assessed using the MTT colorimetric assay. At each time point the absorbance value at 570 nm was detected. The data are shown as a percentage of proliferating cells, per experimental condition. The 0.1% saponin detergent was used as a positive control, ethanol was used as a negative control for HyT. Results are shown as mean ± SEM, two-way ANOVA, **** *p* < 0.0001.

The OMWW A009 extracts were provided by the “Azienda Agricola-Fattoria La Vialla”, following purification as described below. Four batches from different preparations were characterized for molecular contents [10,15,16], revealing similar molecules (Supplementary Table S1, Figure 1). The two most recent batches produced, termed L3 and L4 respectively, were used in this study. Hydroxytyrosol (HyT), purity ≥ 98%, was purchased from Sigma Aldrich (Sigma-Aldrich, Saint Luis, MO, USA) and used as a reference oil polyphenol. For treatments, HyT was dissolved in 100% ethanol (EtOH) and diluted to the same concentration present in A009 in RPMI 1640 medium. Medium with EtOH alone at the same dilution was used as control vehicle for HyT. Crystal violet staining solution and MTT (3-(4,5-dimethylthiazol-2-yl)-2,5 diphenyltetrazolium bromide were purchased from Sigma Aldrich (Milan, Italy).

### 2.2. Cell Culture and Maintenance

Preliminary experiments were conducted on four NSC lung cancer cell lines A459, H1650, H1299, H1975 obtained from ATCC. After observing a similar regulation of growth, (Supplementary Material Figure S2) all the in vitro studies were performed on the A549 and H1650 lung cancer cell lines. All cell lines used were routinely checked for possible mycoplasma contaminations. Cells were cultured and maintained in in RPMI 1640 medium (Euroclone, Pero, MI, Italy) supplemented with 10% fetal bovine serum (FBS, Gibco, Thermo Fisher Scientific, Rodano, MI, Italy), 2 mM l-glutamine (Sigma-Aldrich, Saint Louis, MO, USA), 100 U/mL penicillin, and 100 µg/mL (Sigma-Aldrich, Saint Louis, MO, USA). The cell media used did not contain sodium pyruvate to avoid the generation of oxygen reactive species, resulting from the interaction sodium pyruvate/polyphenols within the A009 extracts.

### 2.3. Preparation of A009 Extracts and Determination of the Phenolic Composition

Olive extract was obtained from OMWW by two sequential crossflow filtration process. In the first step, a ceramic microfiltration (MF) was performed, using 2 tubular membranes in alumina oxide with a 300 KDa cut-off 3 (TAMI membranes, Nyons, France) and a filtering surface of 0.35 m^2^. This allows the removal of solid particles and residual plant particles. The MF permeate was then concentrated, by reverse osmosis (RO) in a Polyamide spiral wound module (Microdyn Nadir, Wiesbaden, Germany) with a filtering surface of 7 m^2^. The RO permeate represents ultrapure water and was discarded. Finally, the RO concentrate, obtained reaching a volume concentration ratio (VCR) of 3.6, constituted the olive extract (here termed A009). Quantification of the phenolic presence in A009 extracts was performed using a high-performance liquid chromatography (HPLC) analysis. The abundance of the polyphenols contained in different batches has been shown in a previous publication [10,15,16] and is detailed in Supplementary Table S1 and Supplementary Figure S1.

### 2.4. Determination of Cell Proliferation In Vitro by Crystal Violet Assay

The effects of the A009 extracts on lung cancer cell proliferation was determined by the crystal violet assay. A549 and H1640 cells (3 × 10^3^ cells/well) were plated into a 96-well plate. Following cell adhesion, fresh complete medium with decreasing dilutions (ranging from 1:50 to 1:5000) of A009 (L4 or L3) or HyT (same dilution present in the A009 extracts) were added for 24–72 h. EtOH dissolved in the RPMI complete medium was used as control vehicle. As positive control, 0.1% of saponin (Sigma Aldrich) was used. Following treatments, plates were washed and incubated with 50 μL of crystal violet staining solution/well, for 20 min at room temperature, gently washed with distilled water, and air-dried for at least 2 h at room temperature. Cell-retained crystal violet was dissolved in 100 μL of crystal violet elution buffer (50% of ethanol and 0.1% acetic acid). Cell viability was measured by detecting the absorbance (595 nm wavelength), with the SpectraMax M2 (Molecular Devices, Sunnyvale CA). Each experimental point was assessed in 12 wells of a 96 well plate. The assay was repeated twice, using 12 replicates per condition.

### 2.5. Determination of Cell Proliferation In Vitro by MTT Assay

The effects of A009 on cell proliferation, as a consequence of mitochondrial disfunction, was assessed by the MTT (3-(4,5-dimethylthiazol-2-yl)-2,5 diphenyltetrazolium bromide) assay. A549 and H1640 cells (3 × 10^3^ cells/well) were plated into a 96-well plate. Following cell adhesion, fresh complete medium with decreasing dilutions (ranging from 1:50 to 1:5000) of A009 (L4 or L3) or HyT (same dilution present in the A009 extracts) were added for 24 to 72 h. EtOH dissolved in the RPMI complete medium was used as control vehicle. As positive control, 0.1% of saponin (Sigma Aldrich) was used. At each time point, media were replaced with fresh RPMI complete medium, supplemented with 10 μL/well of 5 mg/mL MTT reagent, then incubated at for 3 h, 37 °C, 5% CO_2_. Formazan crystals, generated during MTT incubation, were solubilized in a 100 μL/well with pure DMSO. Cell viability was determined by detecting the absorbance (570 nm wavelength), with the SpectraMax M2 (Molecular Devices, Sunnyvale CA). Each experimental point was assessed in 12 wells of a 96 well plate. The assay was repeated twice, using 12 replicates per condition.

### 2.6. Detection of Apoptosis In Vitro

A549 and LT1460 (1.5 × 10^6^ cells/well) cells were treated with A009 (L4 or L3, dilution 1:100, 1:250, 1:500) or HyT (same dilution present in the A009 extracts), for 28 and 48 h. EtOH alone served as control vehicle. Induction of apoptosis was determined by Propidium Iodide (PI, 1 μg/mL) (Sigma Aldrich) and Annexin-V-APC (Immunotools) staining, followed by flow cytometry analysis, using a BD FACS CantoII flow cytometer. Flow data were analyzed with the FACSDiva 6.1.2 software (Becton Dickinson (BD), San Jose, CA, USA) and the FlowLogic (Miltenyi Biotec, Bergisch Gladbach, Germany) software. Experiments were done in duplicate. 

### 2.7. Sprouting Assay In Vitro

The ability of the A009 extracts to limit the formation of invasive structures in vitro, was determined by the sprouting assay on Matrigel layers. 1 × 10^5^ H1640 cells/mL were seeded on a 10 mg/mL of Matrigel (Corning) polymerized layer, in a 96 well plate, and treated for 48 h with the A009 extracts (L4 or L3, dilutions 1:250, 1:500) or HyT (at the same dilution present in the A009 extracts). Micro-photographs of formed beads, w/wo sprouts, were captured using an Axio Vison Rel microscope (Leica) at 10× magnification. The same magnification is reported for each well. The number of sprouts/beads were quantified and counted using the ImageJ software, v 1.52. Assays were run in quadruplicates.

### 2.8. Adhesion Assay In Vitro

The ability of the A009 extracts to interfere with lung cancer cell adhesion was investigated by adhesion assay on fibronectin. Following well coating with 2 μg/mL of fibronectin as in [10]. Then, 2 × 10^4^ A549 or H1650 cells/well, pre-treated with A009 extracts or Hyt as above, were seeded on a 48-well plate. Following 90 min incubation at 37 °C, 5% CO_2_, the supernatants were removed, and cells were washed with PBS and incubated with 100 μL of crystal violet staining solution/well for 20 min at room temperature, gently washed with distilled water, and air-dried for at least 2 h at room temperature. Cell-retained crystal violet was dissolved in 100 μL of crystal violet elution buffer (50% of ethanol and 0.1% acetic acid). Cell adhesion efficiency (proportional to the quantity of crystal violet retained by adherent cells) was measured by detecting the absorbance (595 nm wavelength) with the SpectraMax M2 (Molecular Devices, Sunnyvale CA, USA). Assays were performed 2 times in triplicates. 

### 2.9. Determination of Cytokine/Chemokine Production In Vitro

The effects of the A009 extracts on pro-angiogenic factor production by lung cancer cell lines, was investigate by flow cytometry. A549 and H1650 cells (1.5 × 10^6^ cells/well) were exposed to A009 extracts (L4 or L3, dilutions 1:250, 1:500) or HyT (at the same dilution present in the A009 extracts), for 24 h. EtOH alone served as control vehicle. Following treatments, A549 and H1650 cells were detached and used for flow cytometry. For surface antigen detection, cells were detached and stained for 30 minutes at 4 °C with the monoclonal Phycoerythrin (PE)-conjugated anti-human CXCR4. For intracellular cytokine detection, cells were collected and fixed/permeabilized using the Cytofix/Cytoperm kit from BD Biosciences. Cells were then stained with the following monoclonal PE-conjugated anti-human antibodies to detect the following intracellular antigens: VEGF, CXCL8, CXCL12, CCL2. Cytokine production determined by flow cytometry, using a BD FACS CantoII analyzer. Following doublets exclusion (FSC-H/FSC-A), the PE signal intensity was detected on FSC-A/SSC-A viable-gated cells. Flow data were analyzed using the BD FACSDiva (v6.1.2). Experiments were done in duplicate.

### 2.10. Western Blot Analysis

We assessed the A009 extract ability to target STAT3 phosphorylation, as a biochemical consequence of their cancer preventive and angiopreventive properties. Following 24 h of treatment with A009 extract (1:250, batch 4 or 3) or HyT alone at the same concentration present in A009 dilutions, the cells were lysed in RIPA buffer, supplemented with protease and phosphatase inhibitor cocktails (Roche Diagnostics GmbH). Proteins (50 μg) were separated on the NupageNovex on 4–12% Bis-Tris Gel (Life Technologies) and transferred to a PVDF membrane Amersham Hybond (GE Healthcare Biosciences). Membranes were incubated overnight at 4 °C with anti-p-STAT3 (Tyr705) (Cell Signaling Technology) and with peroxidase-linked anti-rabbit IgG secondary antibodies (GE Healthcare Life science) for 1 h at room temperature. Specific protein bands were detected with Pierce ECL Western Blotting Substrate (ThermoFisher Scientific). Band intensity (revealed as optical density-OD) was quantified using ImageJ software, v 1.52. Every band was normalized versus the respective housekeeping (HK) protein (beta-actin for A549 cells and tubulin for H1650 cells). Finally, HK normalized bands, were further normalized versus not-treated (NT) cells.

### 2.11. Statistical Analysis

The statistical significance between multiple datasets was determined by one-way and two-way ANOVA using GraphPad Prism 7 and 8. Data are expressed as means ± SEM.

## 3. Results

### 3.1. The A009 Extracts Interfere with Cell Proliferation in Lung Cancer Cell Lines

We investigated the ability of two different batches of A009 extract, L3 and L4, to limit cell proliferation in cancer cell lines A549, H1650, H1299, and H1975 obtained from ATCC. After preliminary assessment (Supplementary Figure S2), further analyses were performed on two lung cancer cell lines, A549 and H1650. Hydroxytyrosol, the most abundant polyphenol present in the extracts, was used as a reference compound. We observed that A009 extracts exhibit anti-proliferative activities in a dilution and time-dependent manner, on all cancer cell lines: A549 and H1650 lung cancer cells shown Figure 1A, panels A–D, and all cell lines are shown in Supplementary Figure S1A. We selected the most active A009 1:100, 1:250, and 1:500, batch L3 and L4, for further experiments.

### 3.2. The A009 Extracts Interfere with Cell Proliferation in Lung Cancer Cells Acting on Mitochondria

Based on our results from the crystal violet assay, we investigated whether the anti-proliferative activities ofA009 impacted on cell metabolism by the MTT assay. We observed a dose and time dependent effects of A009, both on A549 and H1650 lung cancer cells (Figure 1B). H1650 cells appears as more sensible to A009 extracts, both for batch 3 and 4, as compared to A549 cells (Figure 1B, panel A–B). Similar effects were induced by HyT, at the same dilution of that present in the A009 extracts, batch 3 and 4 (Figure 1B, panel C). No effects were induced by the control vehicle for HyT (medium with EtOH) (Figure 1B, panel D).

### 3.3. The A009 Extracts Induce Apoptosis in Lung Cancer Cell Lines

**Figure 2:**
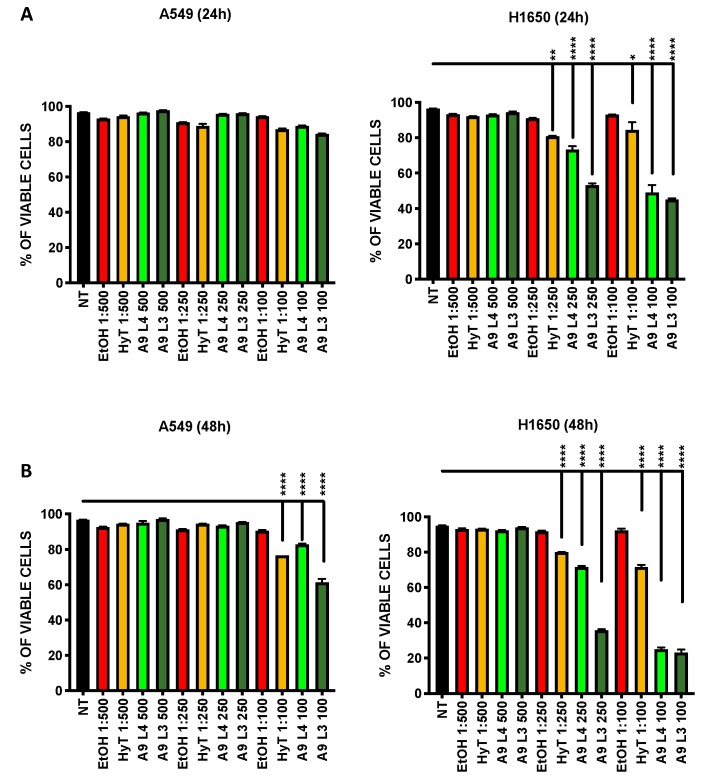
Effect of A009 extracts on the induction of apoptosis in vitro, in lung cancer cells. The lung carcinoma cell lines A549 (A-B) and H1650 (A-B) were treated for 24–48 h with 2 batches of A009 (L4 and L3), at dilutions 1; 500, 1: 250, 1: 100, or with hydroxytytosol (HyT) at the same concentration present in the dilutions of A009. The effects on induction of apoptosis was evaluated by flow cytometry, following labeling with Propidium Iodide (PI) and AnnexinV. Results are shown as mean ± SEM, One Way ANOVA, * *p* < 0.5, ** *p* < 0.01, *** *p* < 0.001, **** *p* < 0.0001. NT (untreated); EtOH (ethanol vehicle used to dissolve hydroxytyrosol).

We tested whether the reduced proliferation of A549 and H1650 lung cancer, following treatment with A009 extracts, was associated with the induction of apoptosis. We found that the A009 extracts were able to induce apoptosis in a dilution and time-dependent manner (Figure 2A,B). Pro apoptotic actives of the of A009 were detected in A549 cells at 48 h (Figure 2, panel B), while significant induction of apoptosis started at 24 h of treatments for H1640 cells (Figure 2, panel A). Following 48 h of treatments, A009 induced apoptosis in a statistically significant manner, starting from 1:250 dilution, and more efficiently as compared to HyT alone at the same dilution present in the A009 extracts (Figure 2B). Based on these results, we selected the dilutions 1:500 and 1:250 for further functional studies.

### 3.4. The A009 Extracts Inhibit Adhesion Capabilities in Lung Cancer Cells

**Figure 3:**
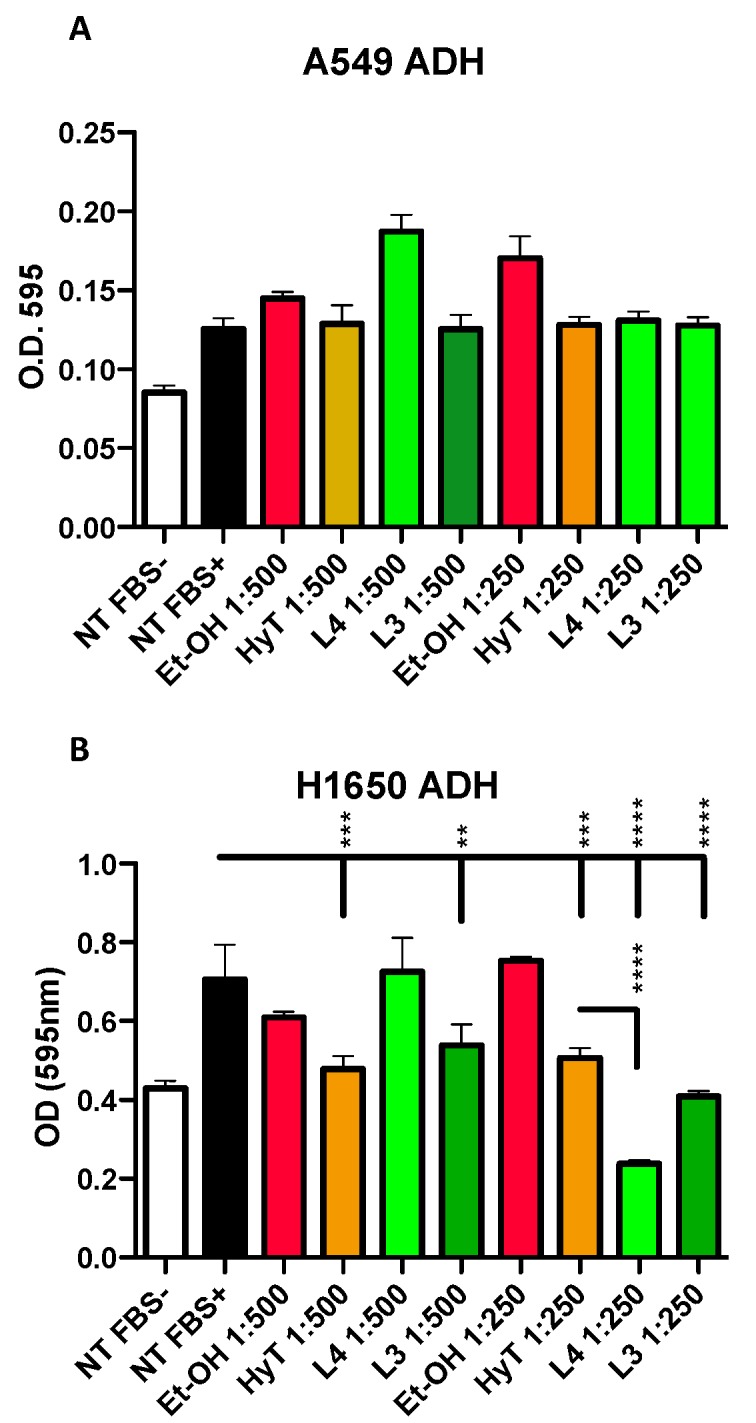
Effect of A009 extracts on cell adhesion in vitro, in lung cancer cells. The lung carcinoma cell lines A549 b (**A**) and H1650 (**B**) were pre-treated, for 24 h, with 2 batches of A009 (L4 and L3), at dilutions 1; 500, 1: 250, or with hydroxytytosol (HyT) at the same concentration present in the dilutions of A009. The effects of A009 on cell adhesion was assessed by seeding pre-treated on a fibronectin layer. Results are shown as mean ± SEM, One Way ANOVA, ** *p* < 0.01, *** *p* < 0.001, **** *p* < 0.0001. NT (untreated); EtOH (ethanol vehicle used to dissolve hydroxytyrosol).

Cell adhesion is a necessary process, together with migration and invasion, for cancer cells to colonize distant sites. Therefore, we tested the capability of the A009 extracts to interfere with lung cancer cells ability to adhere to the fibronectin layer. Interestingly, this is the only property where we found different behavior for the two lung cancer cell lines, A549 and H1650, following exposure to the A009 extracts. H1650 adhesion was reduced by both batches in a dilution dependent manner (Figure 3, panel B) while A549 adhesion properties (Figure 3, panel A) was not affected. 

### 3.5. The A009 Extracts Block Invasive Sprouting in the H1650 Lung Cancer Cells

**Figure 4:**
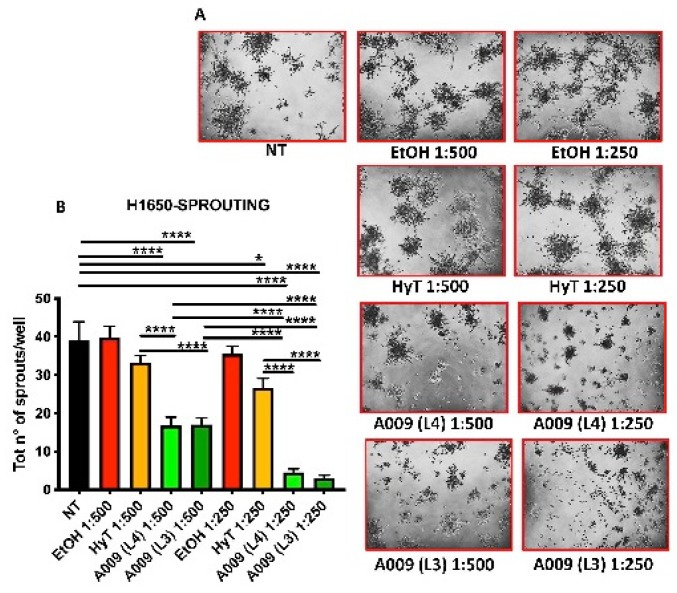
Effect of A009 extract in interfering with pro-metastatic cellular activity in vitro, in lung carcinoma cell. The lung carcinoma cell line H1650 (15.000 cells / well) was seeded in wells containing Matrigel (10 mg / mL) and treated for 48 h with 2 batches of A009 (L4 and L3), at dilutions 1:500, 1: 250, or with hydroxytytosol (HyT) at the same concentration present in the dilutions of A009. The same magnification is reported for each well (10x objective). The effect on the formation of invasive structures (sprouts) (**A**) was determined by counting the absolute number of branches present for each cell spheroid, using ImageJ image analysis software (**B**). Results are shown as mean ± SEM, One Way ANOVA, * *p* < 0.5, **** *p* < 0.0001. NT (untreated); EtOH (ethanol vehicle used to dissolve hydroxytyrosol and diluted to the same levels).

Cell invasion requires the induction of cytoplasmatic elongations necessary for extravasation and colonization of distant sites, during metastasis. Given the selective effects of OMWW on adhesion for H1650 we determined the ability of the A009 extracts to interfere also with H1640 capabilities to produce invasive sprouting on Matrigel (morphologically elongated structures, termed sprouts). We observed that treatments of H1650 cells with A009 extracts reduced the formation of sprouts in a dilution-dependent manner (Figure 4, panels A–B). The inhibition of sprouting was more efficient by using the L4 and L3 A009 extracts, as compared to HyT alone, at the same concentration present in the A009 dilutions (Figure 4, panels A–B).

### 3.6. The A009 Extracts Act on the CXCR4/CXCL12 Axis in Lung Cancer Cell Lines

**Figure 5:**
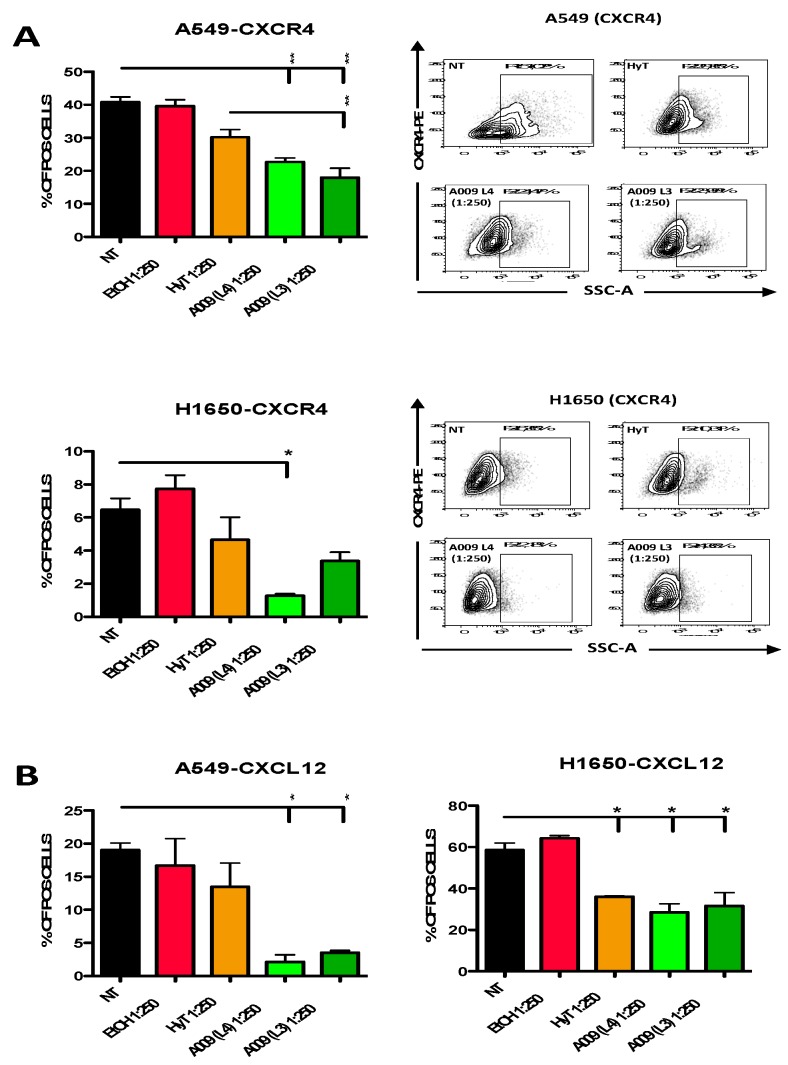
Effect of the A009 extracts on the CXCR4/CXCL12 axis in lung cancer cells in vitro. The A549 and H1650 lung cancer cells were treated for 6 h with 2 different batches of A009 (L4 and L3), at the dilutions 1: 250 or with hydroxytytosol (HyT) at the same concentration present in the dilutions of A009. Protein secretion was stimulated with a cocktail containing BrefeldinA and Monensin + Ionomycin and Forbol 12-meristate, 13-acetate. After 6 h of stimulation, cells were analyzed for CXCR4 (**A**), CXCL12 (**B**) expression, by flow cytometry. Representative dot plots for CXCR4 surface detection are showed (**A**). Flow data were analyzed using FlowLogic software. Results are shown as mean ± SEM, One Way ANOVA, * *p* < 0.5, ** *p* < 0.01. NT (untreated); EtOH (ethanol vehicle used to dissolve hydroxytyrosol).

The CXC4/CXCL12 is a key axis in the acquisition of high motility and invasive properties in cancer cells [18,19,20]. Therefore, we investigated whether the potential chemopreventive abilities of A009 extracts act by modulating of the CXC4/CXCL12 axis. We found that the A009 extracts (batch L4 and L3) significantly inhibit CXCR4 expression and CXCL12 production in A549 and H1650 cells (Figure 5A and Figure 5B) in a dilution-dependent manner. For both the L3 and L4 A009 batches, the effects observed were comparable or greater than those exerted by HyT alone, at the same dilutions present in the A009 extracts.

### 3.7. The A009 Extracts Exert Potential Angiopreventive Properties by Limiting Pro-Angiogenic Cytokine Production in LungCcancer Cell Lines

**Figure 6:**
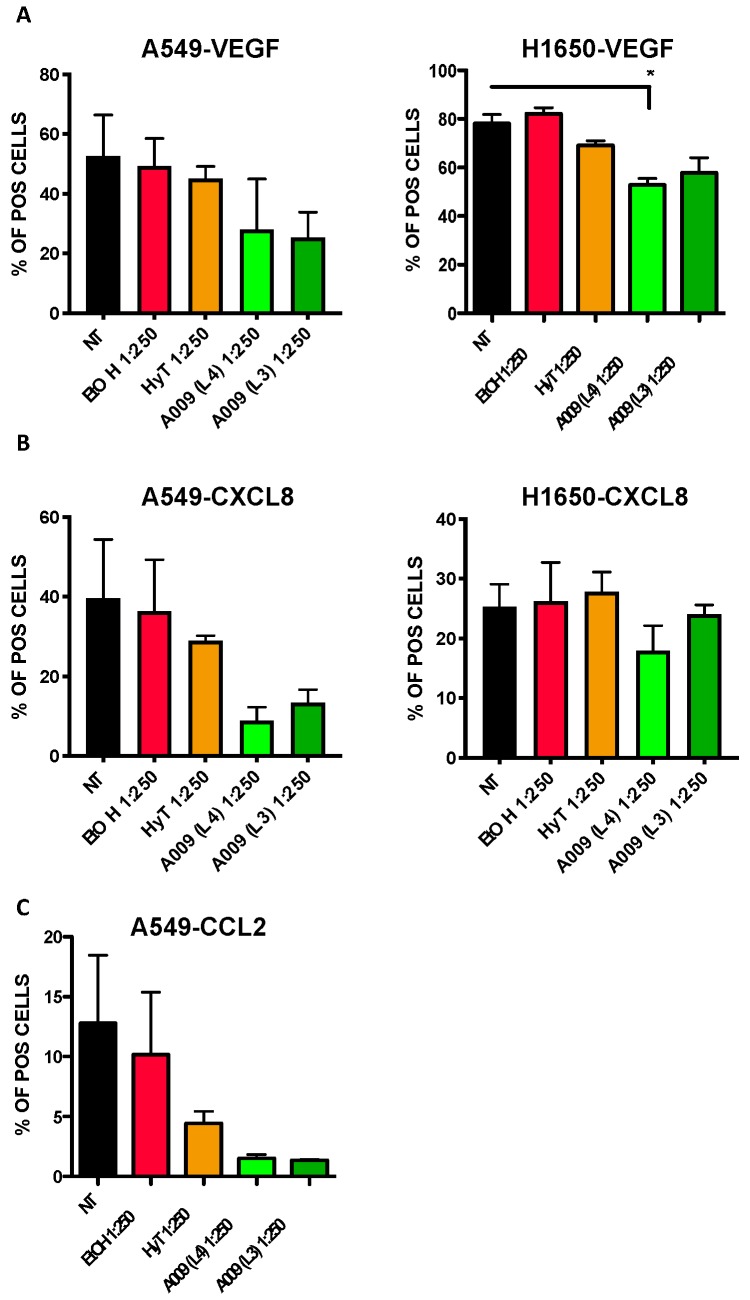
Effect of the A009 extracts on the production of pro-angiogenic factors in lung cancer cells in vitro. The A549 and H1650 lung cancer cells were treated for 6 h with 2 different batches of A009 (L4 and L3), at the dilutions 1; 500, 1: 250 or with hydroxytytosol (HyT) at the same concentration present in the dilutions of A009. Protein secretion was stimulated with a cocktail containing BrefeldinA and Monensin + Ionomycin and Forbol-12-meristate, 13-acetate. After 6 h of stimulation, analyzed for VEGF (**A**), CXCL8 (**B**) and CCL2 (C) production, by flow cytometry. Flow data were analyzed using FlowLogic software. Results are shown as mean ± SEM, One Way ANOVA, * *p* < 0.5. NT (untreated); EtOH (ethanol vehicle used to dissolve hydroxytyrosol).

We investigate whether the A009 extracts exert angio-preventive properties by blocking the production of pro-angiogenic cytokines in lung cancer cell lines. Twenty-four hours of treatment with A009 extracts resulted in decreased production of VEGF and CXCL8 in A549 and H1650 cells (Figure 6, panel A–B) cells and lower production of CCL2 in A549 cells (Figure 6, panel C). Even if the observed trend was not statistically significant, the effects of the A009 extracts, for both batch 4 and 3, was stronger that the effects exerted by HyT alone. 

### 3.8. The A009 Extracts Act on STAT3 Pathway in A549 Lung Cancer Cell Line

**Figure 7:**
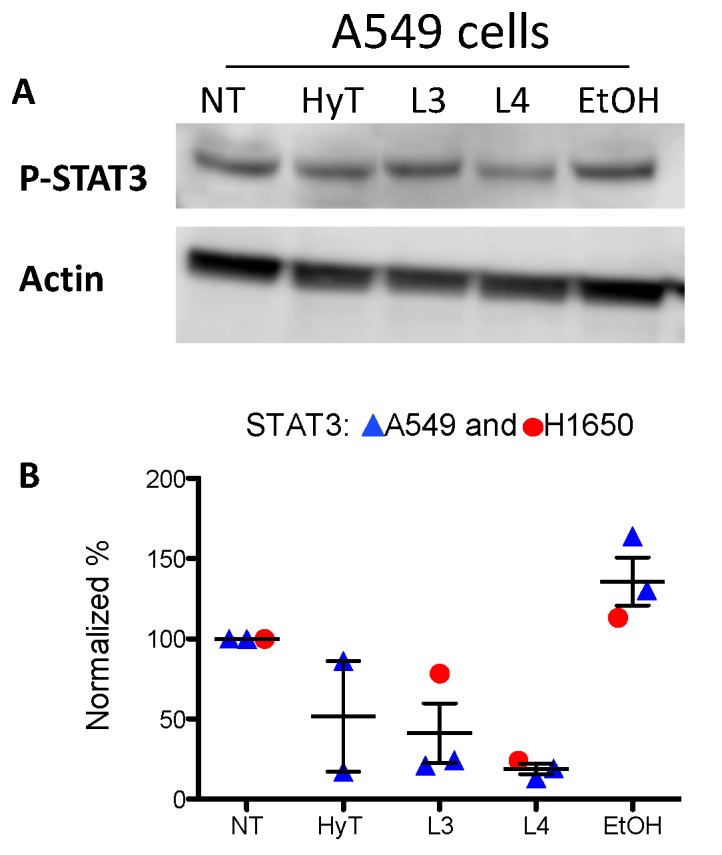
Effects of A009 on STAT3 activation in human lung cancer cells. The A549 cells were treated with two different batches (L4 and L3), 1:250 dilution or HyT, at the same concentration present in A009 dilution for 24 h with A009. Detection of STAT3 phosphorylation (Tyr705) was revealed by western blot analysis. Specific protein band intensity was detected with Pierce ECL Western Blotting Substrate (ThermoFisher Scientific). Protein expressions were normalized to beta-Actin or tubulin. Band intensity (revealed as optical density-OD) were detected by ImageJ software v1.52, and the plot on the lower panel indicates the of levels p-STAT3 in the various conditions. Quantification of results are shown in panel B.

We investigated whether A009 extracts could act on STAT3 pathway, a major hub in the onset of tumor progression. We observed that A009 extracts, 1:250 dilution, batch 4 and 3 can reduce the phosphorylation of STAT3 (Tyr705) on A549 and H1650 lung cancer cells (Figure 7). HyT was used in comparison. 

## 4. Discussion

Cancer, together with cardiovascular diseases, still represents the major cause of deaths worldwide [1,21]. Strong efforts have been made in the identification of successful therapies, alone or in combination with chemotherapy, radiotherapy, as well as targeted and immunotherapies. However, chemoresistance, toxicity, and poor prognosis often lead to the progression of lung and colon cancer, as well as several other neoplasms. In this complex scenario, prevention still represents the best approach. Dietary regimens are now recognized as a real-life approach for cancer prevention, considering that proper foods can be envisaged as a source of relevant molecules, endowed with beneficial effect on human health [22,23]. Adherence to the Mediterranean diet has been largely demonstrated to be associated with the prevention of several chronic-inflammatory disorders, ranging from cardiovascular diseases, neurological disorders, and cancers. EVOO is a major component of the Mediterranean diet and its beneficial effects on health, have been largely investigated and demonstrated [24]. Therefore, olive oil production generates a large quantity of waste materials, with a consequent impact on production costs and the management of environmental health. Interestingly, these waste materials are enriched in polyphenols, endowed with antioxidant, anti-tumor, and anti-angiogenic properties [10].

Two different batches of A009 extract, L3 and L4 were able to limit cell proliferation in four lung cancer cell lines. After preliminary assessment, further analyses were performed on two lung cancer cell lines, A549 (the most resistant to A009) and H1650. Hydroxytyrosol, the most abundant polyphenol present in the extracts, was used as a reference compound. 

Anti-proliferative activity was confirmed by the crystal violet and MTT assay. Part of the reduced proliferation was associated with the induction of apoptosis. We tested the capability of the A009 extracts to interfere with lung cancer cell adhesion as a necessary process for migration and invasion. H1650 adhesion was affected by both batches in a dilution dependent manner. A549 adhesion properties were not affected, indicating that different properties might be involved in the modulation of cancer-associated properties in lung cells. 

The acquisition of a migratory and pro-invasive phenotype is a crucial step for tumor cells to escape from the primary site and pursue the colonization of distant organs [25]. The acquisition of metastatic features is accompanied by morphological changes in the primary tumor cells [26]. Sprouting, referred to as the ability of cells to produce elongated structures, represents a morphological changes during the evasion and colonization of distant organs [27]. We observed that H1650 lung cancer cells, characterized by an invasive phenotype, are able to generate fewer sprouts when exposed to the A009 extracts and this effect was stronger than that observed in cells treated with HyT alone. We then moved to the identification of the possible mechanisms governing these effects on both H1650 and A549 lung cancer cells.

Several studies have demonstrated that the CXCR4/CXCL12 axis plays a crucial role in organ-specific metastasis formation [28,29,30]. CXCL12, a ligand for CXCR4, is abundantly produced by neighboring stromal cells and activation of CXCR4-expressing cancer cells by CXCL12 leads to enhanced chemotaxis, trans-endothelial migration, and invasion. Furthermore, high concentrations of CXCL12 are present at the common sites of metastases, suggesting that CXCL12/CXCR4 signaling may have a role in the homing of cancer cells to distant organs. CXCR4 has been also proposed as a relevant target in lung cancers [31]. We found that treatment of lung cancer cells with the A009 extracts resulted in decreased expression of CXC4 in A549 cells and decreased production of CXCL12 in A569 and H1650 cells. HyT administered alone was also effective when compared with both the L4 and L3 bathes of the A009 extracts.

Angiogenesis in a necessary event for tumor proliferation and dissemination in diverse cancer types, including lung cancers [32,33,34]. Due to a peculiar inflammatory environment, tumors are able to release large amounts of cytokines that induce endothelia cell recruitment and activation within the tumor mass. Among these, VEGF and CXCL8 acts as major orchestrators for endothelial cell activation and recruitment [34,35,36]. Based on our previous results on the angiopreventive properties of the A009 extracts [10], we tested the ability of A009 to limit the production of pro-angiogenic factors in A549 and H1650 lung cancer cells. We have preliminary evidence that two different bathes of A009, sharing HyT as the most abundant polyphenols, reduce the production of VEGF and CXCL8 in A549 and H1650 lung cancer cells, and the production of CCL2 in A549 lung cancer cells.

The signal transducer and activator of transcription (STAT) family of proteins are activated in response to, and mediate the downstream signaling of growth factors and cytokines. STATs are dysregulated in a broad range of cancer types [37,38]. Several cancer-preventive and naturally occurring agents have been reported to target STAT3 molecule. Here, we found that A009 extracts were able to decrease the phosphorylation levels of STAT3 in lung cancer cells, as a potential biochemical mechanisms of cancer prevention.

Finally, the A009 extracts exhibit similar or even stronger effects, when compared to HyT alone, once again suggesting that different polyphenols act synergistically, improving their single agent effects in lung cancer cell lines.

## 5. Conclusions

Our study showed the promising chemopreventive activities of polyphenol-rich extracts from OMWW (A009) in lung cancer cells in vitro, acting on molecular axis governing pro-metastatic features in lung cancer. Our data place A009 as a possible candidate dietary mixture of olive derived supplements for cancer prevention. Advantages of the use of this product as compared to EVOO include: less caloric, low fat, water soluble and therefore potentially drinkable, and ecological, allowing to utilize a waste material recovered for possible nutraceutical use and contributing towards greater environmental health.

## Supplementary Materials

The following are available online at https://www.mdpi.com/2072-6643/12/4/903/s1, Figure S1: Representative full chromatograms for A009 extracts, Figure S2: Effect of the A009 extracts on lung cancer cell proliferation in vitro, Table S1: Phenolic compositions in different A009 extracts.
