# Supplementary material for: An Extract of Olive Mill Wastewater Downregulates Growth, Adhesion and Invasion Pathways in Lung Cancer Cells: Involvement of CXCR4
Source: Nutrients. 2020 Mar 26;12(4):903. doi: 10.3390/nu12040903 (PMC7231173; doi:10.3390/nu12040903)
Supplement: Supplementary file 1 [file nutrients-12-00903-s001.pdf]

Supplementary Materials: The following are available online at www.mdpi.com/xxx/s1, Figure S1: title, Table S1: title, Video S1: title.**:** The following are available online at www.mdpi.com/xxx/s1, Figure S1: title, Table S1: title, Video S1: title.

PHENOLIC COMPOUND	A009 L1 (g/l)	A009 L2 (g/l)	A009 L3 (g/l)	A009 L4 (g/l)
Hydroxytyrosol glucoside	ND	2.01	1.69	1.91
Hydroxytyrosol	2.7	2.52	5.72	5.50
Tyrosol	0.2	0.80	ND	0.69
Chlorogenic acid	0.12	0.10	0.10	0.13
b-Hydroxyverbascoside isomer 1	0.35	0.29	0.14	0.23
b-Hydroxyverbascoside isomer 2	0.32	0.26	0.17	0.23
Verbascoside	0.84	1.04	1.32	1.07
Caffeoyl ester of secologanoside	ND	0.66	0.20	0.23
Oleouropein aglycon	ND	0.71	0.22	0.21
6’-p-Coumaroyl secologanoside	ND	0.38	0.40	0.35
Rutin	0.11	ND	ND	ND
Luteolin-7-o-glucoside	0.22	ND	ND	ND

Supplementary Table S1. Phenolic compositions in different A009 extracts. Quantification of the phenolic presence in four different A009 batches (L1, L2, L3, L4) was performed using a high-performance liquid chromatography (HPLC) analysis.

Representative full chromatogram A009 L3

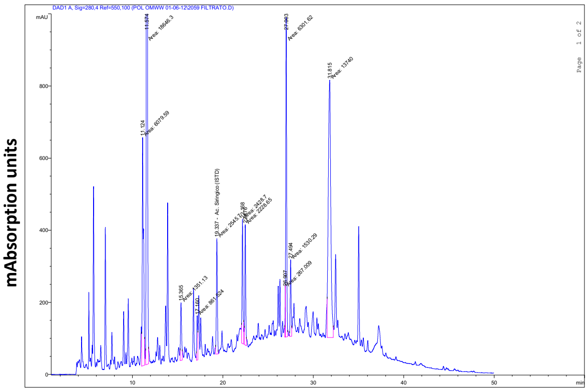

Representative full chromatogram A009 L4

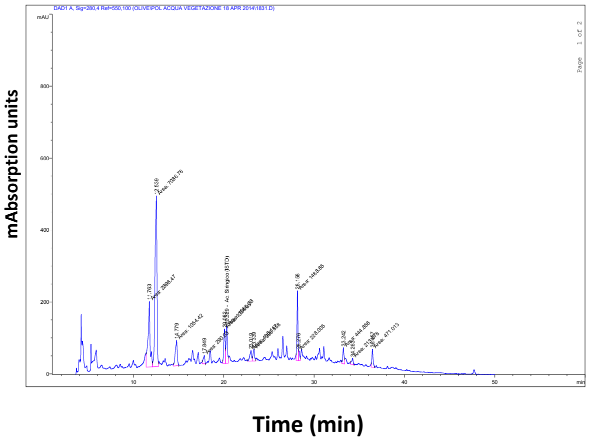

Supplementary Figure S1. Representative full chromatograms for A009 extracts. The full chromatograms for two different batches of A009, L3 and L4, are shown.

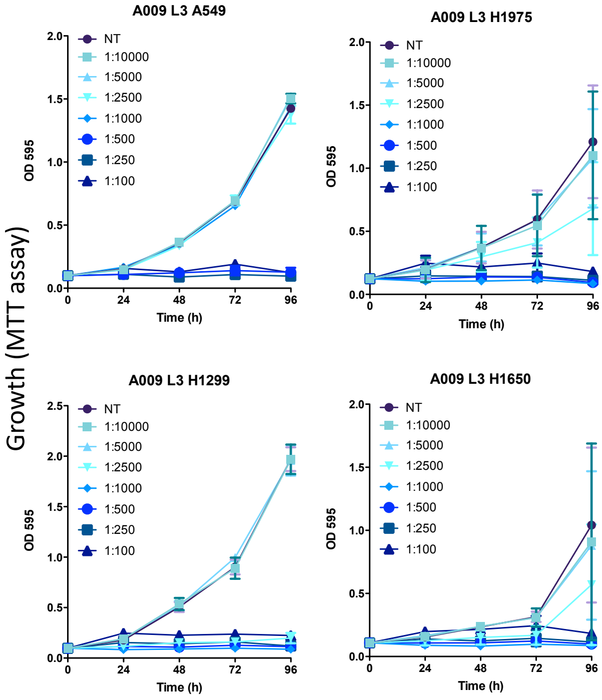

Supplementary Figure S2. Effect of the A009 extracts on lung cancer cell proliferation *in vitro*. Lung carcinoma cell lines were treated for 24-48-72-06 hours the A009 extract (L3), at dilution range 1:10000 to 1:50. The effect on cell proliferation was assessed using the MTT colorimetric assay. At each time point the absorbance value at 570 nm was detected. The data are shown as a percentage of proliferating cells, per experimental condition. Results are shown as mean ± SEM, two-way ANOVA
